# Psychosocial and behavioral health indicators among immigrant and non-immigrant recent mothers

**DOI:** 10.1186/s12884-022-04937-z

**Published:** 2022-08-26

**Authors:** Roheema Ewesesan, Mariette J. Chartier, Nathan C. Nickel, Elizabeth Wall-Wieler, Marcelo L. Urquia

**Affiliations:** 1 Department of Community Health Sciences, Rady Faculty of Health Sciences, Max Rady College of Medicine, University of Manitoba, Winnipeg, MB Canada; 2 Manitoba Centre for Health Policy, University of Manitoba, Winnipeg, MB Canada; 3 Dalla Lana School of Public Health, University of Toronto, Toronto, Ontario Canada

**Keywords:** Maternity, Perinatal, Postpartum, Immigrant, Canada, Depression, Social isolation, Violence, Health behaviors, Prenatal care

## Abstract

**Background:**

Perinatal risk factors can vary by immigration status. We examined psychosocial and behavioral perinatal health indicators according to immigration status and immigrant characteristics.

**Methods:**

We conducted a population-based cross-sectional study of 33,754 immigrant and 172,342 non-immigrant childbearing women residents in Manitoba, Canada, aged 15–55 years, who had a live birth and available data from the universal newborn screen completed within 2 weeks postpartum, between January 2000 and December 2017. Immigration characteristics were from the Canadian federal government immigration database. Logistic regressions models were used to obtain Odds Ratios (OR) with 95% confidence intervals (CI) for the associations between immigration characteristics and perinatal health indicators, such as social isolation, relationship distress, partner violence, depression, alcohol, smoking, substance use, and late initiation of prenatal care.

**Results:**

More immigrant women reported being socially isolated (12.3%) than non-immigrants (3.0%) (Adjusted Odds Ratio (aOR): 6.95, 95% CI: 6.57 to 7.36) but exhibited lower odds of depression, relationship distress, partner violence, smoking, alcohol, substance use, and late initiation of prenatal care. In analyses restricted to immigrants, recent immigrants (< 5 years) had higher odds of being socially isolated (aOR: 9.04, 95% CI: 7.48 to 10.94) and late initiation of prenatal care (aOR: 1.50, 95% CI: 1.07 to 2.12) compared to long-term immigrants (10 years or more) but lower odds of relationship distress, depression, alcohol, smoking and substance use. Refugee status was positively associated with relationship distress, depression, and late initiation of prenatal care. Secondary immigrants, whose last country of permanent residence differed from their country of birth, had lower odds of social isolation, relationship distress, and smoking than primary migrants. There were also differences by maternal region of birth.

**Conclusion:**

Immigrant childbearing women had a higher prevalence of social isolation but a lower prevalence of other psychosocial and behavioral perinatal health indicators than non-immigrants. Health care providers may consider the observed heterogeneity in risk to tailor care approaches for immigrant subgroups at higher risk, such as refugees, recent immigrants, and those from certain world regions.

## Introduction

Maternal, child, and perinatal outcomes may differ by immigration status [1, 2]. The healthy immigrant effect describes the phenomenon by which immigrants exhibit better health outcomes than the receiving-country population. The phenonmenon, which occurs shortly after arrival [3–5], presumably due to selective migration, has been referred to as an explanation for the favorable outcomes of immigrants. On the other hand, immigrants also exhibit substantial heterogeneity across multiple dimensions of vulnerability [6], and the healthy migrant hypothesis may not apply to specific subgroups, such as refugees [7]. Moreover, mixed results in the literature comparing immigrants and non-immigrants also depend on methodological characteristics, such as different study designs, data sources, population diversity and composition, comparison groups, sample sizes, and differences in variable definitions [8–11].

Disparities in perinatal outcomes may also be due to differences in risk factors between immigrants and non-immigrants. The migration process can be considered a psychosocial stressor [12] that can intersect with immigrant womens’ socioeconomic marginalization and vulnerability in the new country [13]. Pregnancy and the perinatal period are sensitive to environmental influences that can trigger and exacerbate complex health risks associated with perinatal psychosocial changes and adjustments [14]. For instance, poor relationship quality or marital stress related to post-migration and acculturation stress [15] may be risk markers for partner violence [16, 17]. The experience of partner violence or abuse can trigger or worsen depression [10, 18, 19] and increase maternal stress levels, which can strongly influence adopting unhealthy coping behaviors, such as smoking and/or alcohol consumption [20, 21]. Unhealthy behaviors have been reportedly associated with social isolation [22] and delayed prenatal care utilization [22, 23].

Among immigrants, variation in perinatal outcomes and risk factors may be driven by immigrant characteristics [2]. Unlike primary immigrants, who immigrate directly from their country of birth, secondary immigrants (also referred to as two-step or serial migrants) reside in at least one intermediate country before immigrating to their final destination [24, 25]. Secondary (voluntary) immigrants are a highly selected subgroup of immigrants characterized by higher educational credentials and global upward socioeconomic mobility, which are conducive to better health outcomes [26]. However, the secondary migration advantage may not apply to refugees who spent long periods of time in refugee camps in intermediate countries [7]. Several studies have documented the erosion of immigrants’ initial health advantage with increasing duration of residence [4, 27, 28], although some health outcomes such as depression may improve with time since migration [29, 30].

Reports examining multiple psychosocial and behavioral perinatal health indicators among immigrant groups are scarce and limited to non-population-based studies with small sample sizes [1, 31, 32]. A qualitative study examined health behaviors (smoking and alcohol use), social support, and stress during pregnancy among seventeen Southeast Asian immigrant women in Montreal, Canada [33]. Aside from a few population-based studies with reported findings by the duration of residence [34] and nativity [35], there is a lack of studies assessing psychosocial and behavioral perinatal health outcomes among immigrant subgroups defined according to refugee status, secondary migration and maternal birth region.

To advance knowledge on the psychosocial and behavioral perinatal health indicators among childbearing immigrant women, we used population-based provincially funded screening data collected in the home by public health nurses, typically within 2 weeks postpartum, linked to Canadian federal government immigration records. All new permanent residents to Canada, including economic and refugee applicants and their dependents are eligible for the free-of-charge publicly-funded universal health care coverage, which includes physician and hospital services that is provincially administered. Some temporary residents (work permit visas) are also eligible for the provincial health care coverage. A small proportion of refugee claimants (also known as asylum seekers) are not covered by the Manitoba Health Care Insurance Plan but by a federally-funded program (i.e., the Interim Federal Health Program) while awaiting resolution of their case. Our objective was to compare select psychosocial and behavioral perinatal health indicators between immigrants to Manitoba, Canada and non-immigrants overall and according to key immigrant characteristics, such as refugee status, secondary migration, maternal birth region, and duration of residence.

## Methods

### Study design, settings, and participants

The population-based cross-sectional study was conducted among childbearing women in Manitoba, Canada. We included immigrant and non-immigrant women aged 15–55 years, who had a live birth between January 1, 2000, to December 31, 2017, and had Baby First or Family First Screening data, a universal newborn screen completed within 2 weeks postpartum.

### Data sources

Multiple de-identified linkable administrative databases were accessed at the Manitoba Population Research Data Repository that contains multi-sectoral information of all Manitoba residents. Upon registration to the Manitoba Health Insurance Plan, the publicly-funded health care coverage for all residents, all individuals are registered in the Manitoba Health Insurance Registry (MHIR) and assigned a Personal Health Identification Number (PHIN) that is used by health care providers for billing purposes. The MHIR contains personal and sociodemographic information. All health and social services databases are linked to the MHIR at the Ministry of Health. After linkage, the databases are de-identified by stripping personal identifiers such as names and addresses and replacing the PHIN by a scrambled unique identifier (SCRPHIN) that allows tracking of individuals across datasets. These de-identified datasets are then made available for research at the Repository [36]. The primary data sources were the Baby First (2000–2002) and Family First (2003–2017) screening data (BFS/FFS). The BFS/FFS screenings were designed to collect data on biological, social, and demographic risk factors of childbearing women in Manitoba [37]. Using the screening form, visiting public health nurses attempt to assess all families with newborns at home within a week of discharge from the hospital. This universal newborn screen provides valuable information for determining appropriate resources and services for the family and population-level surveillance. Information on immigration characteristics was obtained from the Immigration, Refugees, and Citizenship Canada Permanent Resident (IRCC-PR) database, which provides information on the immigration characteristics of all immigrants who obtained permanent residence in Canada from January 1985 to December 2017. The linkage rate of the IRCC-PR and the MIHR was 96% [38]. Additional linked databases were the Discharge Abstract Database from the Canadian Institute for Health Information (for hospital births), the Midwifery Summary System (for home births), and the Social Allowances Management Information Network (SAMIN) database that was used to determine receipt of Employment and Income Assistance (EIA). Small area Census data was used to assign neighborhood income quintiles and rural residence.

### Cohort formation

**Fig. 1:**
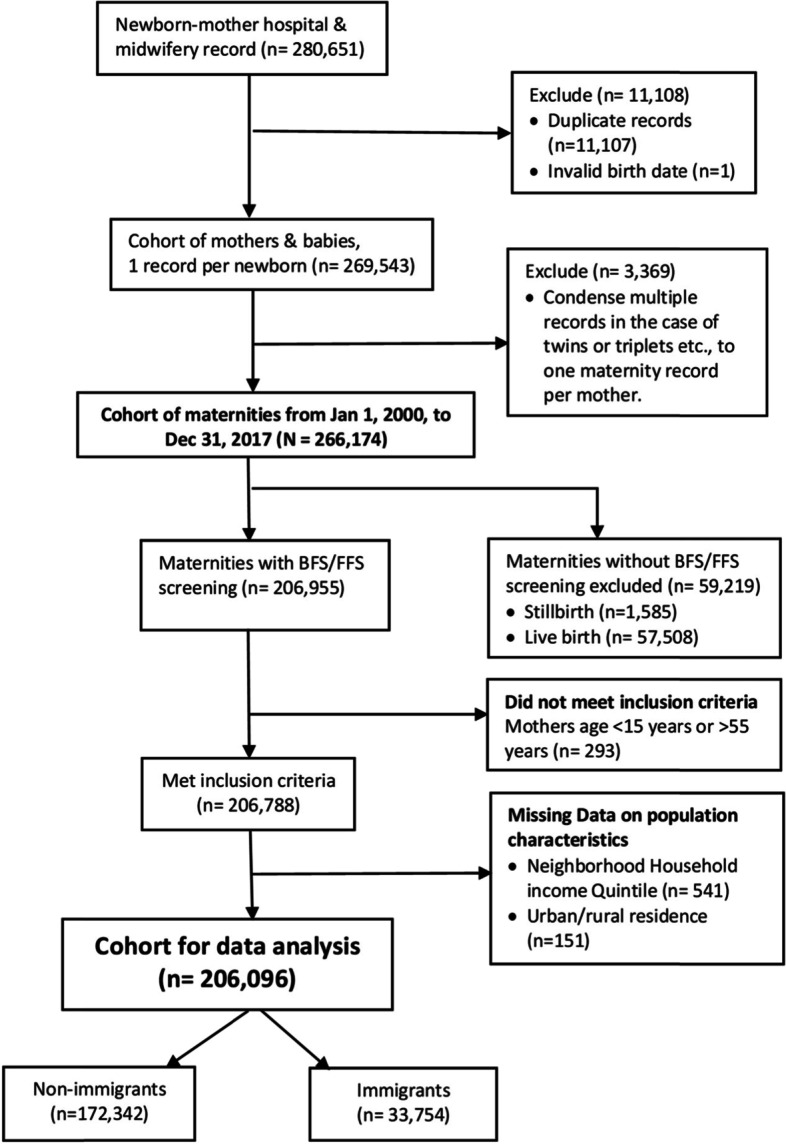
Flow chart cohort selection

Birth records from the Discharge Abstract Database and the Midwifery Summary System from January 1, 2000, to December 31, 2017, were used to create a cohort (*n* = 269,543) of eligible mothers. Cohort included all maternity records, irrespective of the number of offspring or delivery events to a mother over the study period. We reduced multiple records (*n* = 3369) in the case of twins, triplets, or more to one record per maternity and retained *n* = 266,174. We also excluded maternity records not linkable to the BFS/FFS dataset 59,219 (22.2%). These were stillbirths or births resulting in neonatal death and births in which the mother did not participate in the screening due to not being contacted or refused. We further excluded births to mothers aged < 15 years or > 55 years (*n* = 293), those with unknown neighborhood income quintile (*n* = 541), and unknown urban/rural residence (*n* = 151) (Fig. 1).

### Dependent variables

*Psychosocial and behavioral Outcomes* were nurse-recorded information collected during the BFS/FFS. Outcomes examined are *maternal depression,* defined as self-reported depression, during pregnancy up until the postpartum interview (yes, no); *social isolation,* defined as lack of social support or isolation related to culture, language, or geography (yes, no); *relationship distress,* defined as self-reported distress or conflict between parenting partners (e.g., separations, frequent arguments) (yes, no); *partner violence*, defined as self-reported current or history of violence between parenting partners (yes, no); *alcohol use during pregnancy*, defined as self-reported consumption of alcoholic beverages during pregnancy (yes, no); *smoking during pregnancy*, defined as self-reported smoking during pregnancy (yes, no); *substance use during pregnancy*, defined as self-reported illicit drug or substance use during pregnancy (yes, no); and *late initiation of prenatal care*, defined as prenatal care (PNC) that begins after the first trimester of pregnancy (more than 13 weeks gestation) (yes, no). Due to varying number of missing values in the dependent variables, the sample size varied by outcome.

### Explanatory variables

*International immigrants* were identified by having a record in the IRCC-PR database. Women not in the IRCC-PR database and not having a record in the registry indicating that they came from another country were classified as Non-immigrant (Canadian-born). Since the IRCC-PR database began in 1985, a very small portion of immigrants who obtained permanent residence before 1985, when immigration was less common, may have been misclassified as non-immigrants.

*Secondary immigrants* were defined as immigrants to Canada whose last country of permanent residence differed from their country of birth. Those whose countries of birth and last permanent residency were the same were classified as primary immigrants.

*Refugees* were protected persons in Canada or dependants abroad of a protected person in Canada. They include all refugee categories (i.e., Syrian, Government-Assisted Refugees, Privately Sponsored Refugees, and Blended Visa Office-Referred refugees).

*Birth region* was based on the country where the woman was born. Countries were grouped into world regions of birth based on the United Nations classification [39] and regrouped where sample sizes are small as Southeast Asia, South & Rest of Asia, Western Europe, Eastern Europe, Rest of Europe, West Africa, East Africa, Rest of Africa, North America & Oceania, Latin America & the Caribbean.

*Duration of residence* was estimated as the length of time from the date of obtaining permanent residence in Canada to the birth of the child and grouped into < 5 years, 5–9 years, and ≥ 10 years. Some immigrants may have held temporary resident status and had birth events before obtaining permanent residence.

### Covariates

Measured covariates included sociodemographic variables such as mothers’ age (in years) at the birth of the index child (below 20, 20–24, 25–29, 30–34, 35–39, 40–55), low education defined as reported mothers low education or completed <12th grade (yes, no) and lone mother (yes, no) from the BFS/FFS data, urban/rural residence, neighborhood income quintile (Q1- lowest, Q2, Q3, Q4, Q5- highest) from Census data, and receipt of EIA 1 year before birth to 2 weeks after (yes, no) from the SAMIN data. Family factor variables were family size, i.e., the number of dependents < 18 years in the same family as index child (none, at least one) from the MHIR. In analyses restricted to immigrants, knowledge of official Canadian languages (English/French, none) was obtaimed from the IRCC-PR database.

### Statistical analysis

There were missing values in the dependent variables. Individuals with missing values in the dependent variables were excluded based on the assumption that missingness would not strongly bias comparisons between immigrants and non-immigrants [40]. Hence, the final sample size varied for each outcome. There was no percentage thresholds for missing exclusions. Instead, we compared the propotion of missings between immigrants and non-immigrants using standardized differences, with values < 0.1 indicating significant differences. For selected covariates with missing values, we created a dummy value ‘unknown’ to reduce sample size loss [41].

Participants’ characteristics were described using frequencies, percentages, means, and standard deviations. Logistic regression was used to estimate unadjusted (OR) and adjusted odds ratios (aORs) with 95% confidence intervals (CI) for the associations between immigration status and the dependent variables. Because in the BFS/FFS dataset 52% of the maternities were to women who had more than one maternity in the study period, their outcomes are correlated and may affect the associations because of their repeated measurements in the dataset, which violates the assumption of independence of observations [42]. Generalized Estimating Equations (GEE) were therefore used to account for the clustering of maternities within women [43].

In the analyses restricted to immigrants, we used internal comparison subgroups. Primary immigrants were used as the reference group to assess associations by secondary migration, based on studies that found higher rates of psychological distress among secondary immigrants [44]. Similarly, non-refugees were the reference group for analysis that examined associations by refugee status based on higher rates of psychosocial risk factors among women of refugee background [45]. Western Europeans were the reference group for associations by birth region based on their low rates of adverse birth outcomes among European-born women [2, 46], and for being more ethnically alike to the mainstream Canadian-born population. Given that the association of duration of residence and adverse birth outcomes produced mixed results [28], we used 10 or more years of residence as the reference group because of the documented convergence of health status of immigrants with that of the non-immigrant population. Cell counts of less than six were deemed unreportable and suppressed following Manitoba privacy regulations. All analyses were conducted using SAS V9.4 (SAS Institute Inc., Cary, NC).

## Results

Table 1 describes the population characteristics of 172,342 non-immigrant and 33,754 international immigrant cohorts. Immigrant women were slightly older, their average age in the early thirties, and only about 4% were lone mothers compared to 12% among non-immigrants. About 79.3% had higher education or had completed more than high school education, and the majority (78.6%) lived in an urban area. Immigrants were overrepresented (29.9%) in the lowest neighborhood income quintile, but only 4% received employment and income assistance, compared to 16% among non-immigrants. Excluding the index child, 61% of families had one or more other dependent(s) less than 18 years. Most immigrant participants originated from Asia, while for other regions, the proportion ranged from 3.3% for West Africa to 8.6% for Eastern Europe.

**Table 1:** Characteristics of the maternities with BFS/FFS records in Manitoba, Canada, 2000–2017 (*n* = 206,096)

	Non-immigrant 172,342 (83.62%)	International immigrant 33,754 (16.38%)
**Sociodemographic characteristics**		
Mother’s age at birth of index child – mean (SD)^a^	28.20 (5.61)	30.48 (5.33)
Mother’s age at birth of index child – Category		
Below 20 years	11,437 (6.64)	537 (1.59)
20–24 years	34,745 (20.16)	4145 (12.28)
25–29 years	53,935 (31.30)	9786 (28.99)
30–34 years	48,852 (28.35)	11,489 (34.04)
35–39 years	19,958 (11.58)	6243 (18.50)
40–55 years	3415 (1.98)	1554 (4.60)
Lone mother		
Yes	20,116 (11.67)	1280 (3.79)
No	145,045 (84.16)	31,854 (94.37)
Unknown	7181 (4.17)	620 (1.84)
Low education^b^		
Yes	31,974 (18.55)	3362 (9.96)
No	118,781 (68.92)	26,766 (79.30)
Unknown	21,587 (12.53)	3626 (10.74)
Neighborhood Income Quintile		
Q1 (lowest)	35,783 (20.76)	10,089 (29.89)
Q2	35,820 (20.78)	6887 (20.40)
Q3	34,772 (20.18)	6453 (19.11)
Q4	3598 (20.08)	5822 (17.25)
Q5 (Highest)	31,369 (18.20)	4503 (13.34)
Urban/ Rural residence		
Rural	72,881 (42.29)	7224 (21.40)
Urban	99,461 (57.71)	26,530 (78.60)
Received Employment & Income Assistance one year before birth to 2 weeks after		
Yes	27,104 (15.73)	1402 (4.15)
No	145,238 (84.27)	32,352 (95.85)
**Family factor characteristics**		
Family size (Number of dependents < 18 years in the same family as index child)		
None	73,876 (42.87)	13,080 (38.75)
At least one	98,466 (57.13)	20,674 (61.25)
**Immigrant characteristics**		
International Immigrant		
Primary	–	27,362 (81.11)
Secondary	–	6374 (18.89)
Refugee		
Yes	–	4123 (12.22)
No	–	29,613 (87.78)
Knowledge of official Canadian languages (English or French)		
Yes	–	22,074 (65.43)
No	–	11,662 (34.57)
Duration of residence		
< 5 years	–	18,497 (54.83)
5-9 years	–	8782 (26.03)
10 above years	–	6457 (19.14)
Maternal Birth Region		
Southeast Asia	–	9622 (28.51)
South Asia & Rest of Asia	–	10,303 (30.52)
Eastern Europe		2907 (8.61)
Western Europe	–	1356 (4.02)
Rest of Europe	–	1293 (3.83)
East Africa	–	1958 (5.80)
West Africa	–	1097 (3.25)
Rest of Africa	–	1422 (4.21)
North America & Oceania	–	1207 (3.58)
Latin America & Caribbean	–	2589 (7.67)

Frequencies expressed as n (%) unless otherwise specified

^a^*SD* Standard Deviation

^b^Low education = reported mothers’ low education or completed <12th grade

Table 2 presents the different outcome prevalences and the unadjusted and adjusted odds ratios comparing international immigrants to non-immigrants. Missing data in the dependent variables ranged from 4.6% for smoking during pregnancy to 32.4% for partner violence. Only social isolation was more prevalent among immigrants, and the association became stronger after adjustment. Conversely, immigrants had a lower prevalence of the other indicators before and after adjusting for sociodemographic and family factors.

**Table 2:** Prevalence and odds ratio estimates of perinatal health indicators between immigrant and non-immigrant childbearing women in Manitoba, Canada

	International Immigrants		Non-immigrants		International immigrants versus Non-immigrants	
	N	n (%)	N	n (%)	OR (95% CI)^**a**^	aOR (95% CI)^**b**^
**Psychosocial indicators**						
Depression, Mood & Anxiety Disorder (*n* = 181,189)^c^	29,256	1522 (5.20)	151,933	28,022 (18.44)	**0.24 (0.23, 0.26)**	**0.26 (0.25, 0.29)**
Social isolation (*n* = 161,666)^c^	29,428	3605 (12.25)	132,238	4013 (3.03)	**4.46 (4.24, 4.69)**	**6.95 (6.57, 7.36)**
Relationship distress (*n* = 182,438)^c^	29,971	642 (2.14)	152,467	9155 (6.00)	**0.34 (0.31, 0.37)**	**0.72 (0.66, 0.78)**
Partner violence (*n* = 139,234)^c^	24,686	126 (0.51)	114,548	2716 (2.37)	**0.21 (0.18, 0.25)**	**0.51 (0.42, 0.62)**
**Behavioral indicators**						
Alcohol use during pregnancy (*n* = 164,723)^**c**^	29,983	868 (2.89)	134,740	19,069 (14.15)	**0.18 (0.17, 0.19)**	**0.23 (0.21, 0.25)**
Smoking during pregnancy (*n* = 196,623)^**c**^	32,547	584 (1.79)	164,076	32,123 (19.58)	**0.08 (0.07, 0.08)**	**0.11 (0.10, 0.12)**
Substance use during pregnancy (*n* = 163,898)^**c**^	29,867	66 (0.22)	134,031	6377 (4.76)	**0.04 (0.03, 0.06)**	**0.09 (0.07, 0.11)**
Late initiation of prenatal care > 13 weeks (*n* = 194,202)^**c**^	32,261	301 (0.93)	161,941	3544 (2.19)	**0.42 (0.37, 0.48)**	**0.84 (0.74, 0.95)**

Frequencies expressed as n (%)

^a^*OR* Odds Ratios with 95% CI (Confidence Intervals) are derived from Generalized Estimating Equation models

^b^*aOR* Odds Ratios with 95% CI (Confidence Intervals) are derived from Generalized Estimating Equation models, adjusted for Maternal age, lone mother, low education, neighborhood income quintile, urban residence, family size, and employment & income assistance

Bold values = Significant association (*p* < 0.05)

^c^Total excludes missing values for outcomes where (*n*=)

Frequencies missing for outcomes: (Depression, mood & anxiety disorder n = 24,907; social isolation *n* = 44,430; Relationship distress *n* = 23,658; Partner violence *n* = 66,862; Alcohol use during pregnancy *n* = 41,373; Smoking during pregnancy *n* = 9473; Substance use during pregnancy *n* = 42,198; Late initiation of prenatal care > 13 weeks *n* = 11,894)

Tables 3 and 4 present odds ratio estimates restricted to international immigrant childbearing women. Secondary compared to primary immigrants had a higher prevalence of social isolation, but this association reversed after adjustment (Table 3). Being a secondary immigrant was also negatively associated with relationship distress. Refugees were more likely to experience relationship distress, depression, mood, and anxiety disorder than non-refugees. The high likelihood of social isolation became marginally significant, while partner violence became non-significant in the adjusted analysis. Compared to Western Europe, women from all birth regions were more likely to experience social isolation, except women from North America and Oceania, who had a lower prevalence, although not statistically significant. Only women from West Africa had a high likelihood of relationship distress in both unadjusted and adjusted analyses. Women from African regions were more likely to experience partner violence. The association was attenuated and non-significant in the adjusted analysis but remained high among women from West Africa. Those from North & South Europe, North America & Oceania, Latin America & the Caribbean were more likely to have depression, mood & anxiety disorder. Recent immigrants had nine times the odds of being socially isolated than those with ten or more years of residence but lower odds of relationship distress and depression than long-term immigrants.

**Table 3:** Unadjusted and adjusted odds ratio estimates of psychosocial perinatal health indicators among immigrant childbearing women in Manitoba, Canada

International immigrants *n* = 33,754	Social isolation (*n* = 29,428)^**c**^		Relationship distress (*n* = 29,971)^**c**^		Partner violence (*n* = 24,686)^**c**^		Depression, mood & anxiety disorder (*n* = 29,256)^**c**^	
	OR (95% CI)^**a**^	aOR (95% CI)^**b**^	OR (95% CI)^**a**^	aOR (95% CI)^**b**^	OR (95% CI)^**a**^	aOR (95% CI)^**b**^	OR (95% CI)^**a**^	aOR (95% CI)^**b**^
**Immigrants**								
Primary	1.0 (Reference)	1.0 (Reference)	1.0 (Reference)	1.0 (Reference)	1.0 (Reference)	1.0 (Reference)	1.0 (Reference)	1.0 (Reference)
Secondary	**1.25 (1.13, 1.37)**	**0.76 (0.68, 0.85)**	**0.68 (0.54, 0.86)**	**0.73 (0.55, 0.97)**	0.87 (0.55, 1.36)	0.84 (0.50, 1.43)	0.85 (0.73, 1.00)	0.98 (0.82, 1.18)
**Refugees**								
Non-refugee	1.0 (Reference)	1.0 (Reference)	1.0 (Reference)	1.0 (Reference)	1.0	1.0 (Reference)	1.0 (Reference)	1.0 (Reference)
Refugee	**1.46 (1.32, 1.62)**	1.17 (0.99, 1.38)	**3.70 (3.09, 4.42)**	**1.39 (1.02, 1.90)**	**3.54 (2.37, 5.28)**	1.02 (0.52, 1.98)	**1.70 (1.46, 1.98)**	**1.37 (1.11, 1.70)**
**Maternal birth region**								
Western Europe	1.0 (Reference)	1.0 (Reference)	1.0 (Reference)	1.0 (Reference)	1.0 (Reference)	1.0 (Reference)	1.0 (Reference)	1.0 (Reference)
Southeast Asia	**2.10 (1.50, 2.95)**	**1.76 (1.21, 2.56)**	1.31 (0.79, 2.19)	1.21 (0.68, 2.16)	1.48 (0.52, 4.20)	1.04 (0.35, 3.03)	**0.71 (0.51, 0.98)**	0.71 (0.49, 1.04)
South & Rest of Asia	**4.02 (2.88, 5.61)**	**3.03 (2.10, 4.36)**	0.83 (0.49, 1.40)	1.28 (0.73, 2.26)	1.07 (0.37, 3.08)	1.12 (0.38, 3.30)	0.76 (0.55, 1.06)	0.93 (0.65, 1.34)
Eastern Europe	**3.39 (2.38, 4.84)**	**2.95 (2.01, 4.33)**	0.68 (0.36, 1.28)	0.99 (0.50, 1.95)	1.36 (0.41, 4.49)	1.54 (0.44, 5.34)	0.99 (0.69, 1.45)	1.08 (0.72, 1.61)
Rest of Europe	**1.60 (1.06, 2.42)**	**2.01 (1.30, 3.10)**	1.11 (0.57, 2.13)	1.06 (0.53, 2.10)	1.02 (0.23, 4.57)	0.85 (0.18, 4.07)	**2.39 (1.65, 3.44)**	**2.06 (1.39, 3.07)**
East Africa	**4.37 (3.07, 6.23)**	**2.45 (1.62, 3.69)**	**2.67 (1.54, 4.61)**	1.12 (0.57, 2.21)	**3.18 (1.05, 9.58)**	0.86 (0.22, 3.34)	0.77 (0.51, 1.17)	0.62 (0.38, 1.00)
West Africa	**3.63 (2.50, 5.27)**	**2.59 (1.71, 3.92)**	**3.43 (1.93, 6.08)**	**2.03 (1.02, 4.02)**	**4.77 (1.50, 15.16)**	1.92 (0.54, 6.90)	0.78 (0.48, 1.27)	0.76 (0.45, 1.30)
Rest of Africa	**6.03 (4.21, 8.63)**	**3.59 (2.40, 5.38)**	**3.60 (2.08, 6.23)**	1.51 (0.77, 2.96)	**3.38 (1.05, 10.91)**	0.75 (0.20, 2.91)	1.33 (0.90, 1.95)	1.10 (0.70, 1.73)
North America & Oceania	0.81 (0.50, 1.34)	0.65 (0.38, 1.09)	0.76 (0.37, 1.58)	0.82 (0.38, 1.77)	1.38 (0.34, 5.52)	1.25 (0.32, 4.85)	**3.35 (2.32, 4.82)**	**3.46 (2.33, 5.14)**
Latin America & Caribbean	**4.03 (2.84, 5.74)**	**3.21 (2.20, 4.69)**	**2.57 (1.51, 4.39)**	1.48 (0.83, 2.64)	1.44 (0.44, 4.70)	0.63 (0.19, 2.14)	**2.43 (1.73, 3.42)**	**2.14 (1.47, 3.11)**
**Duration of residence**								
< 5 years	**7.90 (6.61, 9.45)**	**9.04 (7.48, 10.94)**	**0.53 (0.44, 0.65)**	**0.68 (0.53, 0.87)**	0.84 (0.52, 1.36)	1.22 (0.71, 2.10)	**0.39 (0.34, 0.45)**	**0.46 (0.39, 0.53**)
5–9 years	**2.42 (1.99, 2.95)**	**2.64 (2.15, 3.24)**	**0.74 (0.59, 0.91)**	0.84 (0.66, 1.08)	1.12 (0.67, 1.86)	1.32 (0.75, 2.32)	**0.58 (0.50, 0.67)**	**0.65 (0.56, 0.75)**
10 years and more	1.0 (Reference)	1.0 (Reference)	1.0 (Reference)	1.0 (Reference)	1.0 (Reference)	1.0 (Reference)	1.0 (Reference)	1.0 (Reference)

^a^*OR* Odds Ratios with 95% CI (Confidence Intervals) are derived from Generalized Estimating Equation models

^b^*aOR* Odds Ratios with 95% CI (Confidence Intervals) are derived from Generalized Estimating Equation models, adjusted for Maternal age, lone mother, low education, neighborhood income quintile, urban residence, family size, employment & income assistance, and knowledge of official Canadian languages

Bold values = Significant association (*p* < 0.05)

^c^Total excludes missing values for outcomes where (*n*=)

Frequencies missing for outcomes: (Social isolation *n* = 4326; Relationship distress *n* = 3783; Partner violence *n* = 9068; Depression, mood & anxiety disorder *n* = 4498)

**Table 4:** Unadjusted and adjusted odds ratio estimates for behavioral perinatal health indicators among immigrant childbearing women in Manitoba, Canada

International immigrants n = 33,754	Maternal alcohol use during pregnancy (*n* = 29,983)^**c**^		Maternal smoking during pregnancy (*n* = 32,547)^**c**^		Maternal substance use during pregnancy (*n* = 29,867)^**c**^		Late initiation of Prenatal care (*n* = 32,261)^**c**^	
	OR (95% CI)^a^	aOR (95% CI)^b^	OR (95% CI)^a^	aOR (95% CI)^b^	OR (95% CI)^a^	aOR (95% CI)^b^	OR (95% CI)^a^	aOR (95% CI)^b^
**Immigrants**								
Primary	1.0 (Reference)	1.0 (Reference)	1.0 (Reference)	1.0 (Reference)	1.0 (Reference)	1.0 (Reference)	1.0 (Reference)	1.0 (Reference)
Secondary	**0.77 (0.64, 0.94)**	0.94 (0.75, 1.17)	**0.70 (0.53, 0.93)**	**0.71 (0.52, 0.97)**	0.64 (0.31, 1.30)	0.81 (0.37, 1.78)	**1.63 (1.25, 2.13)**	1.32 (0.97, 1.78)
**Refugees**								
Non-refugee	1.0 (Reference)	1.0 (Reference)	1.0 (Reference)	1.0 (Reference)	1.0 (Reference)	1.0 (Reference)	1.0 (Reference)	1.0 (Reference)
Refugee	1.18 (0.96, 1.46)	0.95 (0.72, 1.25)	**1.98 (1.54, 2.53)**	1.20 (0.85, 1.71)	**2.60 (1.43, 4.76)**	0.94 (0.40, 2.19)	**2.43 (1.86, 3.17)**	**1.59 (1.07, 2.36)**
**Maternal birth region**								
Western Europe	1.0 (Reference)	1.0 (Reference)	1.0 (Reference)	1.0 (Reference)	(−)	(−)	1.0 (Reference)	1.0 (Reference)
Southeast Asia	0.86 (0.60, 1.23)	1.02 (0.66, 1.60)	**0.53 (0.34, 0.82)**	0.63 (0.36, 1.11)	(−)	(−)	1.37 (0.65, 2.92)	1.88 (0.82, 4.30)
South & Rest of Asia	**0.31 (0.21, 0.45)**	**0.45 (0.28, 0.71)**	**0.28 (0.17, 0.45)**	**0.40 (0.23, 0.72)**	(−)	(−)	0.96 (0.45, 2.08)	1.23 (0.54, 2.82)
Eastern Europe	1.43 (0.98, 2.09)	**1.96 (1.27, 3.04)**	1.02 (0.64, 1.62)	1.53 (0.89, 2.62)	(−)	(−)	1.78 (0.79, 4.00)	1.77 (0.75, 4.17)
Rest of Europe	**2.39 (1.58, 3.60)**	**2.10 (1.31, 3.39)**	**3.62 (2.28, 5.74)**	**3.57 (2.10, 6.07)**	(−)	(−)	1.30 (0.51, 3.30)	1.54 (0.58, 4.07)
East Africa	**0.61 (0.38, 0.97)**	0.83 (0.46, 1.49)	0.61 (0.35, 1.05)	**0.41 (0.20, 0.86)**	(−)	(−)	1.86 (0.81, 4.26)	1.06 (0.40, 2.76)
West Africa	0.54 (0.29, 1.01)	0.63 (0.31, 1.27)	**0.26 (0.11, 0.61)**	**0.25 (0.09, 0.65)**	(−)	(−)	1.68 (0.67, 4.22)	1.48 (0.53, 4.15)
Rest of Africa	0.68 (0.41, 1.12)	0.84 (0.46, 1.51)	0.53 (0.27, 1.04)	**0.35 (0.14, 0.87)**	(−)	(−)	**2.53 (1.10, 5.81)**	1.40 (0.55, 3.60)
North America & Oceania	**2.00 (1.31, 3.05)**	**2.47 (1.52, 4.02)**	1.48 (0.83, 2.65)	1.64 (0.85, 3.20)	(−)	(−)	1.01 (0.37, 2.74)	1.04 (0.37, 2.93)
Latin America & Caribbean	**1.69 (1.15, 2.47)**	**1.74 (1.10, 2.74)**	1.51 (0.95, 2.41)	1.14 (0.66, 1.99)	(−)	(−)	1.76 (0.78, 3.96)	1.31 (0.56, 3.05)
**Duration of residence**								
< 5 years	**0.35 (0.30, 0.41)**	**0.43 (0.35, 0.52)**	**0.33 (0.27, 0.40)**	**0.46 (0.36, 0.60)**	**0.08 (0.04, 0.17)**	**0.07 (0.03, 0.16)**	1.26 (0.93, 1.72)	**1.50 (1.07, 2.12)**
5–9 years	**0.42 (0.35, 0.51)**	**0.52 (0.42, 0.64)**	**0.43 (0.34, 0.54)**	**0.58 (0.45, 0.75)**	**0.39 (0.23, 0.68)**	**0.44 (0.25, 0.78)**	0.86 (0.59, 1.25)	0.94 (0.64, 1.40)
10 years and more	1.0 (Reference)	1.0 (Reference)	1.0 (Reference)	1.0 (Reference)	1.0 (Reference)	1.0 (Reference)	1.0 (Reference)	1.0 (Reference)

^a^*OR* Odds Ratios with 95% CI (Confidence Intervals) are derived from Generalized Estimating Equation models

^b^*aOR* Odds Ratios with 95% CI (Confidence Intervals) are derived from Generalized Estimating Equation models, adjusted for Maternal age, lone mother, low education, neighborhood income quintile, urban residence, family size, employment & income assistance, and knowledge of official Canadian languages

Bold values = Significant association (*p* < 0.05)

(−) = unreportable (*n* < 6 for internal comparison group)

^c^Total excludes missing values for outcomes where (*n*=)

Frequencies missing for outcomes: (Alcohol use during pregnancy *n* = 3771; Smoking during pregnancy *n* = 1207; Substance use during pregnancy *n* = 3887; Late initiation of prenatal care > 13 weeks *n* = 1493)

Only smoking during pregnancy was lower among secondary immigrants than primary immigrants (Table 4). Refugees had higher prevalences of smoking and substance use, but the associations became non-significant after adjustment. Refugees more likely experienced late initiation of PNC compared to non-refugees. Compared to Western Europeans, pregnancy smoking and alcohol consumption were higher among North & South Europe women in the adjusted analysis. Women from Eastern Europe, North America & Oceania, and Latin America & the Caribbean had higher adjusted odds of consuming alcohol but not of smoking. Those from Africa had lower adjusted odds of smoking but not of alcohol consumption, while those from South & Rest of Asia had significantly lower odds of smoking and alcohol use. We did not report odds ratio estimates for substance use by birth regions because the cell count for the internal comparison group (Western Europeans) was less than six. Late initiation of PNC did not exhibit significant variation by region of birth after adjustment. Recent immigrants were less likely to consume alcohol, smoke, or engage in substance use during pregnancy but had 50% higher odds of late initiation of PNC compared to long-term immigrants.

## Discussion

### Main findings

In our population-based study, immigrants had a higher prevalence of social isolation than non-immigrants but a lower prevalence of other outcomes. Psychosocial and behavioral risks varied by immigrant characteristics; refugee status was strongly associated with relationship distress, depression, and late initiation of PNC; recent immigration with social isolation and late initiation of PNC and certain maternal birth regions with relationship distress, depression, smoking, and/or alcohol use during pregnancy.

### Strengths and limitations

A major strength of this study was a novel linkage of the BFS/FFS data with the immigration data. Another strength is the population-based nature of the study, including a large and ethnically diverse sample. However, our study had limitations. First, the BFS/FFS data had varying degrees of missing data in the dependent variables. Missingness in some dependent variables may have affected some comparisons between immigrants and non-immigrants, given that missingness was higher among non-immigrants (23.3% for social isolation, 33.5% for partner violence, 21.8% for alcohol use and 22.2% for substance use) than among immigrants (12.8% for social isolation, 26.9% for partner violence, 11.2% for alcohol use and 11.5% for substance use), with standardized differences larger than 0.1. There were no statistically significant differences in the proportion of missing values according to immigration status for depression (overall 12.1% missing), relationship distress (11.5%), smoking during pregnancy (4.6%) and late initiation of prenatal care (5.8%). On the other hand, social desirability may have underestimated prevalence of some behavioral indicators such as alcohol and substance use during pregnancy. Similarly, individuals experiencing partner violence are less likely to respond to this question for various reasons, including fear of police involvement. However, although some outcomes may be underreported, associations may only be biased if the underreporting was differential according to immigration status. Overall, the potential impact of missingness on the magnitude of associations is uncertain, but the direction may hold for most of the outcomes, given the strong observed associations.

Second, given that the public health nurses complete the BFS/FFS based on their assessment and the information provided by the mother, interviewer bias cannot be ruled out. Although some recall bias may occur, this is likely minimal because of the short time frame between the birth and the completion of the BFS/FFS. Third, excluding women without screening data (i.e., who gave birth to stillborn or children who died in the early neonatal period or had a live birth but did not participate in the screening) implies that our findings may not be generalizable to these cases. Our results apply to permanent residents and cannot be generalized to temporary residents not covered by the provincial health insurance plan, such as asylum seekers. Our measure of duration of residence may underestimate exposure to the Canadian environment among those who became permanent residents after being temporary residents. Fourth, residual confounding due to unmeasured factors or non-detailed measurements, such as the number of cigarettes per day for smoking or frequency and amount of alcohol consumed, may have affected the associations and efficiency of adjustment. Likewise, our data did not distinguish specific forms of partner violence; physical (assault, battery), sexual, verbal abuse, psychological aggression, or control (financial, emotional, coercion) [47]. Last, we could not measure associations for substance use by maternal birth region due to very small sample sizes for certain birth regions.

Regardless of these limitations, our findings contribute to a greater understanding of the complexity of psychosocial and behavioral perinatal health risks of immigrant childbearing women by identifying patterns and subgroups at higher and lower risk, as follows.

### Social isolation

The prevalence of social isolation among immigrants in our study (12.3%) was similar to the prevalence reported in a sample of German adults [48]. Culturally relevant support often enjoyed in home countries may be lost after migration-related family separation [49] or not well-established in western countries post-migration [50]. This may explain the high odds among immigrants and across all birth regions [31, 51] except North America & Oceania [52], most of whom are native English speakers, which could explain their lower odds. The result for recent immigrants is unsurprising, given that time is needed to integrate into a new environment and rebuild social networks. Establishing multiple networks over time may improve access to appropriate information that promotes positive health behaviors or minimize stressful situations [53] and possibly explain the drastic reduction after 5 years of residence.

### Relationship distress

We found an overall low prevalence among immigrants compared to non-immigrants. A few studies on marital relationships among immigrants are qualitative [15–17] with no comparative results for non-immigrants. Among immigrants, the high odds of experiencing relationship distress for refugees and African immigrant women are consistent with a few qualitative studies [16, 54, 55]. Unlike economic immigrants, refugees’ forced migration may exacerbate disagreements between couples in terms of perceptions and expectations regarding life post-migration. Their disadvantaged social status [56, 57] may create financial hardship, triggering conflict where females depend more on their spouses financially. African men have reportedly shown low involvement and support towards home care or responsibilities [55]. Acculturation stress and gender role reversal [15, 54] can trigger conflict, where women’s new financial power threatens their partner’s authority [58], and women combine home care with work demands. The low prevalence among secondary immigrants is consistent with reported better health outcomes among secondary immigrants who voluntarily transitioned to Canada from an industrialized country compared to primary migrants from non-industrialized birth [24]. A plausible explanation is the selective migration of couples pursuing upward global social mobility [24] that may have gained more economic advantage [26], reducing stressors accompanying their transit [44]. Similarly, selective migration of couples in harmonious relationships may explain the lower odds among recent immigrants.

### Partner violence

Consistent with past studies [59–61] is the low prevalence among immigrants, which may be related to differences in the perception of violence. Some immigrant women may not consider some acts violent, may be reluctant to acknowledge or report violence, or overcome structural barriers to navigating help within a complex immigration system [62]. Cultural or social pressures and financial dependency on spouses may cause them to tolerate abuse in silence [47]. The high odds among women from Africa were of significant interest, particularly those from West Africa. This association was very strong but was no longer significant after adjustment, explained partially by control variables and partially by a relatively small subgroup size. We highlight this result based on reported high rates and risk of physical abuse or partner violence among Africans [58, 63]. Normalization of violence may be due to stigma or fear, particularly among women from regions where women have subordinate roles and are disempowered [64].

### Depression

Although many studies reported a high prevalence of depression among immigrants compared to non-immigrants [18, 19, 30], our findings align with other studies that found a lower prevalence [65], consistent with the healthy migration hypothesis. Our results further confirmed this hypothesis among recent immigrants, consistent with past studies from the United States [66] and Canada [65]. Conversely, other studies have reported high risk among recent migrants [18, 29] and risk regardless of time since migration [65]. The higher odds of depression among refugees align with previous findings [18, 32]. Unlike economic migrants driven by upscale social mobility and selected for migration, refugees are displaced people who did not seek to migrate and are generally less healthy [7]. Our result also agrees with the reportedly high risk among women from Europe [67], Latin America & the Caribbean [30, 68]. Social support is protective against depression [18, 69], and we found that immigrant women were more socially isolated. Being socially isolated may underlie depression among these birth regions, particularly refugees, due to loss of homeplace practices related to childbirth and support post migration [70].

### Alcohol use, smoking & substance/drug use

Our results agree with studies conducted in Canada [22, 23, 34], the United States [35], and France [20] that have shown that immigrants were less likely to engage in unhealthy behavior during pregnancy compared to non-immigrants, which may be related to protective cultural strengths that immigrants bring with them from their countries of origin [71]. We observed similar results among recent immigrants, consistent with a study in the United Kingdom that reported low smoking prevalence that increased for every five-year additional length of stay [72]. The lower odds among secondary immigrants may be due to more health system utilization advantage they likely gained during their transit [26]. Conversely, we found high-risk patterns for consuming alcohol among East Europe, North America & Oceania, and Latin America & the Caribbean, in addition to smoking among North & South Europe, consistent with a Swedish population-based study that found high pregnancy smoking prevalence among immigrant women [73]. Longer historical exposure to cultural acceptance of smoking and alcohol and more gender equality may explain the high prevalence among immigrants from these westernized regions compared to regions where women do not traditionally drink or smoke, as the case is for South & Rest of Asia and Africa.

### Prenatal care

Past studies have demonstrated a high likelihood of late initiation of PNC among immigrants compared to non-immigrants [74–77]. Instead, we found a lower likelihood, which partly could be due to selection bias and may mean that immigrants who completed a screening may be more committed to PNC than those who did not. Protective for late initiation of PNC among immigrants to Manitoba perhaps is access to universal health care and the opportunity to choose from several available care providers such as family physicians, obstetricians, and midwives. However, plausible explanations for the high risk among refugees [78] and recent immigrants [74, 79] may be less familiarity with the receiving country’s health care system, language barriers, insufficient support to access services, and discordant expectations between the women and their service providers [13, 79]. It may also be possible that recent immigrants were underrepresented in the screening since a longer duration of residence was associated with lower odds of late initiation of prenatal care.

## Conclusion

In this population-based study, we found that compared to non-immigrants, immigrant childbearing women exhibited a higher prevalence of social isolation but a lower prevalence of other psychosocial and behavioral perinatal health indicators. Immigrant subgroups such as refugees, recent immigrants, and certain maternal birth regions exhibited high-risk patterns for multiple indicators.

Findings from this study may help health care providers increase awareness of the heterogeneity of the immigrant population and immigrant subgroups at higher and lower risk for psychosocial and behavioral perinatal health outcomes, and incorporate these considerations in the continuum of care. The high risk for social isolation among recent immigrants, multiple perinatal psychosocial risks among refugee immigrants, and other high-risk patterns in behavioral outcomes among particular birth regions may reflect the lack of awareness of settlement resources, expectation mismatch, or unmet needs across the range of currently available services in the province. Our findings also evidence the limitation of the BFS/FFS as a surveillance tool, particularly concerning high proportion of non-response in some key indicators of risk. Findings from this study call for further research towards exploring and identifying unmet needs across new and existing support programs for immigrant childbearing women. Much attention should focus on the growing diversity of immigrants, particularly recent and refugee immigrants.

## Data Availability

The datasets generated and analyzed for this study are not publicly available because of Manitoba privacy regulations for highly sensitive personal information data. Access to data at the Manitoba Population Research Data Repository can be granted to those who meet pre-specified criteria for confidential access. Requests to access data for research purposes must be sent to MCHP_Access@cpe.umanitoba.ca. Refer to MCHP policies on data access and use [80].

## Abbreviations

BFS/FFS: Baby First Screening/ Family First Screening
IRCC-PR: Immigration, Refugees, and Citizenship Canada Permanent Resident
SAMIN: Social Allowances Management Information Network
MHIR: Manitoba Health Insurance Registry
PNC: Prenatal care
